# Author Correction: Dial-in Topological Metamaterials Based on Bistable Stewart Platform

**DOI:** 10.1038/s41598-019-55179-7

**Published:** 2020-01-10

**Authors:** Ying Wu, Rajesh Chaunsali, Hiromi Yasuda, Kaiping Yu, Jinkyu Yang

**Affiliations:** 1 Department of Astronautic Science and Mechanics, Harbin Institute of Technology, Harbin, Heilongjiang 150001 China; 2 Aeronautics and Astronautics, University of Washington, Seattle, WA 98195 USA

Correction to: *Scientific Reports* 10.1038/s41598-017-18410-x, published online 08 January 2018

Four supplementary movies were omitted from the original version of this Article. This has been corrected in the HTML version of the Article; the PDF version was correct at time of publication.

