# Supplementary material for: Dial-in Topological Metamaterials Based on Bistable Stewart Platform
Source: Sci Rep. 2018 Jan 8;8:112. doi: 10.1038/s41598-017-18410-x (PMC5758775; doi:10.1038/s41598-017-18410-x)
Supplement: Supplementary file 1 — Supplementary document [file 41598_2017_18410_MOESM1_ESM.pdf]

Supplementary material: Dial-in Topological Metamaterials Based on Bistable Stewart Platform

Ying Wu,[1,2] Rajesh Chaunsali,[2] Hiromi Yasuda,[2] Kaiping Yu,[1,*] and Jinkyu Yang[2,*]

[1]*Department of Astronautic Science and Mechanics, Harbin Institute of Technology, Harbin, Heilongjiang 150001, China*

[2]*Aeronautics and Astronautics, University of Washington, Seattle, WA 98195, USA*

PACS numbers: 45.70.-n 05.45.-a 46.40.Cd

SUPPLEMENTARY NOTE 1: GENERALIZED MODEL OF SP

Here we show how the conventional SP that has six DOFs of the top plate and a fixed bottom plate can be reduced to a 3-DOF structure by maintaining symmetry in the connecting spring constant attached between two plates. Based on Kane's method[1], we formulate the full dynamic model of 6-DOF SP. These DOFs are labeled as u, v, w, ψ, ϑ, ϕ. Here, u, v, and w denote the displacements, and ψ, ϑ, and ϕ represent the rotations of the top plate along x-, y-, and z-directions, respectively (see Supplementary Fig. 1a). Therefore, the full stiffness matrix of SP is:

$$\mathbf{B}_{full}^{SP} = \mathbf{J}\mathbf{B}_{\Delta}\mathbf{J}^{\mathrm{T}} = \begin{bmatrix} k_{uu} & 0 & 0 & k_{u\psi} & k_{u\vartheta} & 0 \\ 0 & k_{vv} & 0 & k_{v\psi} & k_{v\vartheta} & 0 \\ 0 & 0 & k_{ww} & 0 & 0 & k_{w\phi} \\ k_{\psi u} & k_{\psi u} & 0 & k_{\psi\psi} & 0 & 0 \\ k_{\vartheta v} & k_{\vartheta v} & 0 & 0 & k_{\vartheta\vartheta} & 0 \\ 0 & 0 & k_{\phi w} & 0 & 0 & k_{\phi\phi} \end{bmatrix}, \tag{S1}$$

where, $\mathbf{B}_{\Delta} = diag[\ k_1\ \ k_2\ \ k_1\ \ k_2\ \ k_1\ \ k_2\ ]$ represents the symmetrical arrangement of spring constants connecting two disks, and $\mathbf{J} = \begin{bmatrix} \mathbf{u}_1 & \cdots & \mathbf{u}_6 \\ \tilde{\mathbf{p}}_1\mathbf{u}_1 & \cdots & \tilde{\mathbf{p}}_6\mathbf{u}_6 \end{bmatrix}$ is the Jacobian matrix, in which, $\mathbf{u}_i$ is the unit vector along the direction of ith spring connector, and $\tilde{\mathbf{p}}_i$ is the skew-symmetric matrix for the position vector $\mathbf{p}_i$ of the ith spring joint on the top disk (see Supplementary Fig. 1a). The entries marked in blue denote the displacement and rotation along the z-direction, which are naturally coupled with each other but are independent with the rest four DOFs. As a result, we are able to extract k_{ww}, $k_{w\phi}$, $k_{\phi w}$, and $k_{\phi\phi}$ from the stiffness matrix and add one rotation DOF to the bottom disk to eventually fabricate the stiffness matrice of our simplified 3-DOF SP as

$$\mathbf{B}_{reduced}^{SP} = \begin{bmatrix} k_{\phi\phi} & -k_{\phi\phi} & -k_{w\phi} \\ -k_{\phi\phi} & k_{\phi\phi} & k_{w\phi} \\ -k_{\phi w} & k_{\phi w} & k_{ww} \end{bmatrix}, \tag{S2}$$

with

$$\begin{aligned} k_{ww} &= 3k_1 h_0^2/l_1^2 + 3k_2 h_0^2/l_2^2 \\ k_{\phi\phi} &= 3k_1 R^4[1+\cos(2\theta_0 - 5\pi/3)]/l_1^2 \\ &\quad + 3k_2 R^4 \sin^2(\theta_0 - 2\pi/3)/l_2^2 \\ k_{w\phi} = k_{\phi w} &= 2R^2 k_1 h_0 \sin(\theta_0 - 2\pi/3)/l_1^2 \\ &\quad + 2\sqrt{3}R^2 k_1 h_0 \cos(\theta_0 - 2\pi/3)/l_1^2 \\ &\quad - 2R^2 k_2 h_0 \sin(\theta_0 - 2\pi/3)/l_2^2. \end{aligned}$$

Here, l_1 and l_2 represent the lengths of alternating springs with constants k_1 and k_2 as shown in Supplementary Fig. 1a.

SUPPLEMENTARY NOTE 2: BISTABLE BEHAVIOR ANALYSIS OF SP

To analyze the bistable behavior of the SP unit cell, we can further reduce 3-DOF SP structure to a 2-DOF structure. This is because only the relative height and angle between the two plates determine the stable configurations. So, for simplicity, we can restrict the rotation of the bottom disk and only analyze the rotation and translation of the top disk for this study. As shown in Supplementary Fig. 1a, let the initial height and rotation angle be h_0 and θ_0, respectively (representing the Y-state). We examine the folding behavior of the SP structure by using the principle of minimum total potential energy[2,3]. First, we consider the length of the spring elements l_1 and l_2 as shown in Supplementary Fig. 1a . Based on the geometry of the SP, we can derive l_1 and l_2 as follows (see[2]):

$$\begin{aligned} l_1 &= \sqrt{(h_0 - w)^2 + 4R^2\sin^2\left(\frac{\phi}{2} + \frac{\theta_0}{2} - \frac{\pi}{6}\right)}, \\ l_2 &= \sqrt{(h_0 - w)^2 + 4R^2\sin^2\left(\frac{\phi}{2} + \frac{\theta_0}{2} + \frac{\pi}{6}\right)}. \end{aligned} \tag{S3}$$

From Eq. (S3), we can calculate the deformation in the spring elements and thus the total elastic energy as

$$U = \frac{3}{2}k_1(l_1 - L_1)^2 + \frac{3}{2}k_2(l_2 - L_2)^2, \tag{S4}$$

where, L_1 and L_2 are the initial lengths of the two springs.

Also, the work done on this system is

$$W = Fw + T\phi, \quad \text{(S5)}$$

where, F and T are the external force and torque, respectively.

Then, the total potential energy ($\Pi = U - W$) would simply reduce to the total elastic energy U under no external force/torque in our system, such that

$$\Pi(w,\phi) = U = \frac{3}{2}k_1(l_1 - L_1)^2 + \frac{3}{2}k_2(l_2 - L_2)^2. \quad \text{(S6)}$$

Therefore, applying the principle of minimum total potential energy (i.e., $\delta\Pi = 0$) to Eq. (S6), we have

$$\partial\Pi/\partial w = 0 \quad \text{and} \quad \partial\Pi/\partial\phi = 0, \quad \text{(S7)}$$

which are used to obtain a trajectory to analyze the folding behavior of the SP unit cell.

We plot the total potential energy of SP unit cell during folding/unfolding motion by using Eq. (S6) in Fig. 1b. In the parameter space of w and ϕ, the highlighted region indicates low energy levels. The darkest spots indicate two local minima in the system, and hence, prove the existence of two stable equilibrium states: Y-state and P-state. By further focusing on this highlighted trajectory we deduce a double-well potential curve (Supplementary Fig. 1c) showing the energy level as a function of the normalized displacement. Note that this bistable behavior can be tuned by the geometrical parameters such as the initial height (h_0) and angle (θ_0), as well as the materials properties such as spring constants k_1 and k_2.

Therefore, for the initial stable configuration, i.e., Y-state ($h_0 = 0.04$ m, $\theta_0 = 70°$), we can obtain the parameters for another stable P-state ($h_0 = 0.0215$ m, $\theta_0 = 110°$). In addition, the stiffness matrices for these states would be

$$\mathbf{B}^{Y-state}_{reduced} = \begin{bmatrix} 69.13 & -69.13 & -2.167\times10^3 \\ -69.13 & 69.13 & 2.167\times10^3 \\ -2.167\times10^3 & 2.167\times10^3 & 6.813\times10^4 \end{bmatrix},$$

$$\mathbf{B}^{P-state}_{reduced} = \begin{bmatrix} 3.846 & -3.846 & -268.8 \\ -3.846 & 3.846 & 268.8 \\ -268.8 & 268.8 & 1.969\times10^4 \end{bmatrix}.$$

Judging from the magnitude of the entries, it is clear that the Y-state is stiffer than the P-state.

SUPPLEMENTARY NOTE 3: EIGENVALUE PROBLEM FOR HEXAGONAL UNIT CELL

The unit cell of hexagonal lattice has two SP units, each with three DOFs. Hence, for a wave vector $\mathbf{k}$, 6×6 stiffness and mass matrices in Eq. 3 of the main text are given by

$$\mathbf{B} = \begin{bmatrix} 3k_{cc}+k^{(1)}_{\phi\phi} & -k^{(1)}_{\phi\phi} & -k^{(1)}_{\phi w} & k_{cc}(1+e^{-i\mathbf{k}\cdot\mathbf{a}_1}+e^{-i\mathbf{k}\cdot\mathbf{a}_2}) & 0 & 0 \\ -k^{(1)}_{\phi\phi} & k^{(1)}_{\phi\phi} & k^{(1)}_{\phi w} & 0 & 0 & 0 \\ -k^{(1)}_{w\phi} & k^{(1)}_{\phi w} & k^{(1)}_{ww} & 0 & 0 & 0 \\ k_{cc}(1+e^{i\mathbf{k}\cdot\mathbf{a}_1}+e^{i\mathbf{k}\cdot\mathbf{a}_2}) & 0 & 0 & 3k_{cc}+k^{(2)}_{\phi\phi} & -k^{(2)}_{\phi\phi} & -k^{(2)}_{\phi w} \\ 0 & 0 & 0 & -k^{(2)}_{\phi\phi} & k^{(2)}_{\phi\phi} & k^{(2)}_{\phi w} \\ 0 & 0 & 0 & -k^{(2)}_{\phi w} & k^{(2)}_{\phi w} & k^{(2)}_{ww} \end{bmatrix},$$

and

$$\mathbf{M} = \begin{bmatrix} I & 0 & 0 & 0 & 0 & 0 \\ 0 & I & 0 & 0 & 0 & 0 \\ 0 & 0 & m & 0 & 0 & 0 \\ 0 & 0 & 0 & I & 0 & 0 \\ 0 & 0 & 0 & 0 & I & 0 \\ 0 & 0 & 0 & 0 & 0 & m \end{bmatrix}.$$

To illustrate the analogy with systems exhibiting QVHE, we express the effective Hamiltonian of the system as[4–6]

$$\mathbf{H}(\mathbf{k}) = \mathbf{M}^{-1/2}\mathbf{B}\mathbf{M}^{-1/2} = 3\sigma_0\otimes\tau_0 + \sigma_0\otimes\tau_1 + (c\sigma_1 - s\sigma_2)\otimes\tau_0 - \xi\sigma_3\otimes\tau_1, \quad \text{(S8)}$$

where σ_0, σ_1, σ_2, and σ_3 are the Pauli matrices; $\tau_0 = \tau_{-1/2}\tau_e\tau_{-1/2}$, $\tau_1 = \tau_{-1/2}\tau_k\tau_{-1/2}$, $\tau_{-1/2} = \tau_m^{-1/2}$ are the parameter matrices, in which $\tau_e = diag(k_c, 0, 0)$, $\tau_{-1/2}$ = average of stiffness matrices of Y and P states, $\tau_m = diag(I, I, m)$, $c = 1 + \cos(\mathbf{k}\cdot\mathbf{a}_1) + \cos(\mathbf{k}\cdot\mathbf{a}_2)$, and $s = \sin(\mathbf{k}\cdot\mathbf{a}_1) + \sin(\mathbf{k}\cdot\mathbf{a}_2)$.

The first two terms in the Hamiltonian are the constant matrices that just translate the band dispersion curves without changing their topology. The third term, which is similar to the effective mass Hamiltonian of graphene, leads to the formation of a Dirac cone[6,7]. The last term emerges due to the breakage of the inversion symmetry (thus keeping C_3 symmetry), and it will vanish when the two SPs inside a unit cell are at the same stable configuration (i.e., C_6 symmetry).

SUPPLEMENTARY NOTE 4: ANGLE DEPENDENT WAVEGUIDES

We have already shown in the main text that various complex shaped topological waveguides can be built in the system and a robust wave propagation can be observed. In this note, we highlight a subtle difference in transmission if one uses bends of different angles. In Supplementary Fig. 2, we show topological waveguides at two angles: 120° and 60°, and their traditional counterparts. Note that both waveguides in Supplementary Fig. 2a-b are topological. However, due to the difference in the bend angles, the interface connections are not identical along the waveguides. In the former, the interface all along the length of the waveguide is made of Y-Y connections. But, in the later, it is a mix of Y-Y and P-P connections. These distinct connections can be understood as the direct extension of *soft-soft* and *hard-hard* type topological interfaces in 1D systems[8]. We evaluate the transmission characteristics of these and compare them with their traditional counterparts shown in Supplementary Fig. 2c-d.

Shown in Supplementary Fig. 3 are the transmission profiles for a sinusoidal frequency sweep signal given at the input of the waveguides. One sees that the topological waveguides are clearly superior to traditional waveguides. However, there is not any discernible difference between the transmission of two distinct topological waveguides. This makes sense as this transmission profile is mainly dictated by the presence of local modes inside the band gap, and both the topological waveguides guarantee the presence of local modes all along the band gap.

In Supplementary Fig. 4, we compare these two topological waveguides in transient conditions by sending a 50 ms Gaussian wave packet at 760 Hz. We notice that the waveguide with 120° bend is absolutely back scattering free, however, 60° waveguide leads to a minute back scattering. This is the subtle difference in term of transmission properties of the topological waveguide at different angles, which arises due to the uneven interface along the length of the waveguide as mentioned above.

---

* Corresponding authors: yukp@hit.edu.cn (Kaiping Yu), jkyang@aa.washington.edu (Jinkyu Yang)

[1] Wu, Y., Yu, K., Jiao, J.& Zhao R. Dynamic modeling and robust nonlinear control of a six-DOF active microvibration isolation manipulator with parameter uncertainties. Mech. Mach. Theory **92**, 407-435 (2015).

[2] Reddy, J. Theory and analysis of elastic plates and shells (CRC Press, Boca Raton 2006).

[3] Yasuda, H., Tachi, T., Lee, M.,& Yang, J. Origami-based tunable truss structures for non-volatile mechanical memory operation. arXiv preprint arXiv:1702.05402 (2016).

[4] Lu, J., Qiu, C., Ke, M.& Liu, Z. Valley vortex states in sonic crystals. Phys. Rev. Lett. 116, 093901 (2016).

[5] Lu, J., Qiu, C., Ye, L., Fan, X., Ke, M., Zhang, F. & Liu, Z. Observation of topological valley transport of sound in sonic crystals. Nat. Phys. 13, 369-374 (2017).

[6] Pal, R. K.& Ruzzene, M. Edge waves in plates with resonators: an elastic analogue of the quantum valley Hall effect. New J. Phys. 19, 025001 (2017).

[7] Kane, C. L.& Mele, E. J. Quantum spin Hall effect in graphene. Phys. Rev. Lett. 95, 226801 (2005).

[8] Chaunsali, R., Kim, E., Thakkar, A., Kevrekidis, P. G.& Yang, J. Demonstrating an in situ topological band transition in cylindrical granular chains. Phys. Rev. Lett. **119**, 024301 (2017).

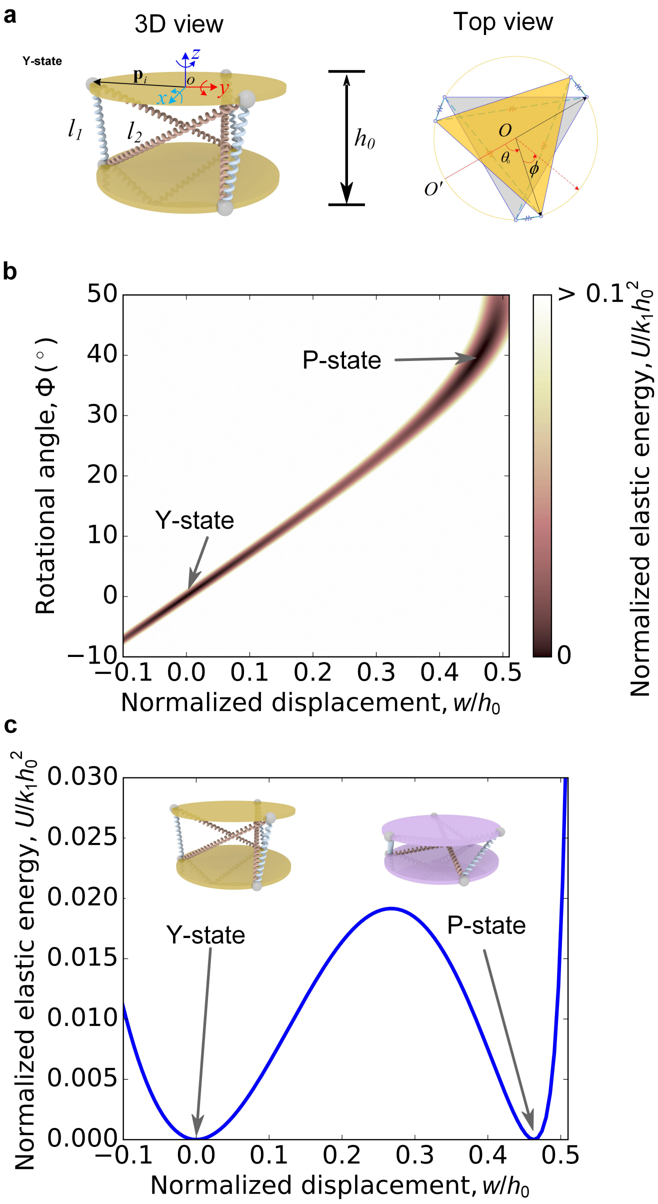

Supplementary Fig. 1: **Bistable behavior of the Stewart Platform (SP).** (**a**) 3D (left) and top (right) views of the SP unit cell are shown. (**b**) The surface map of the elastic energy as a function of both w and ϕ shows the two local minimum states, i.e., Y- and P- states as denoted by arrows. The energy is normalized by $k_1 h_0^2$ and the displacement is normalized by h_0. (**c**) The normalized energy as a function of w is plotted along the minimum potential energy trajectory which corresponds to the highlighted region in (**b**). The inset illustrations show the schematic configuration of the SP at each local minimum state.

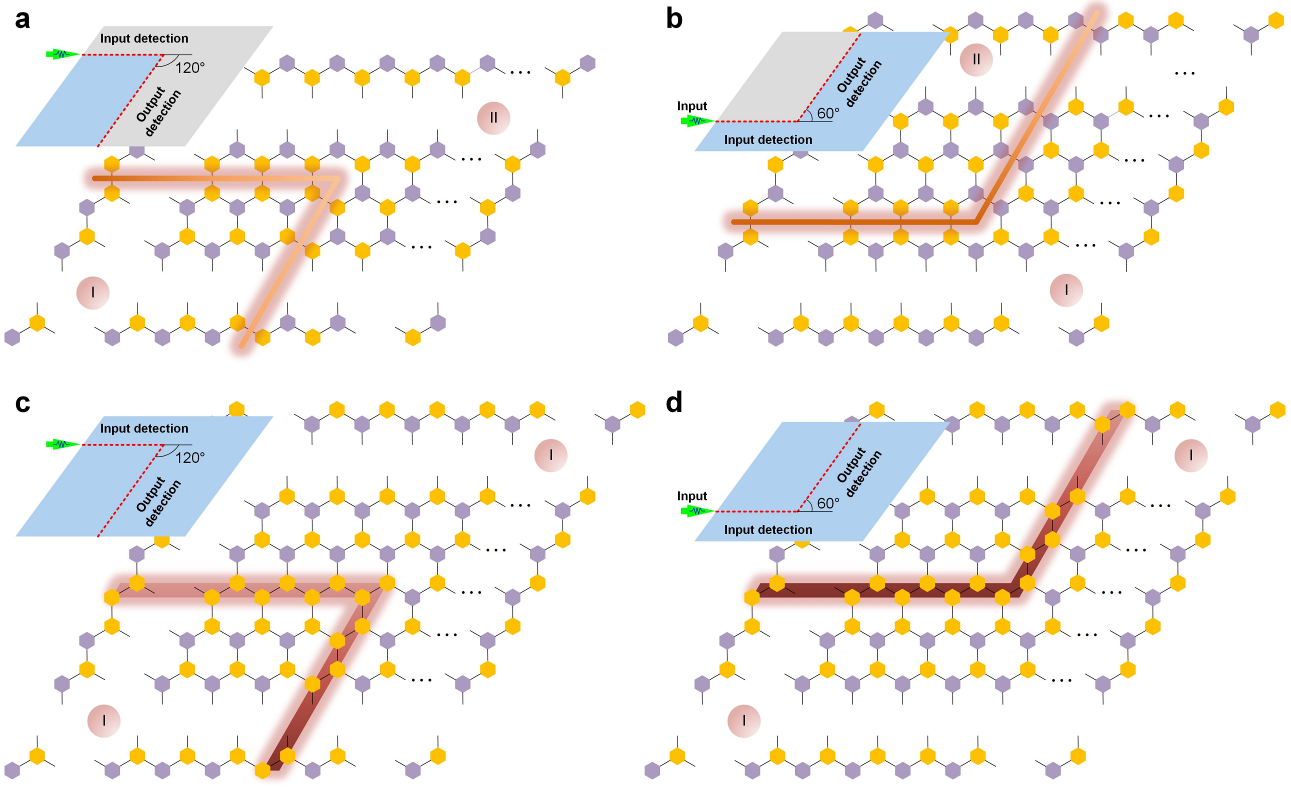

Supplementary Fig. 2: **Topological and traditional waveguides with two different bend angles.** (**a**) and (**b**) are topological waveguides with 120° and 60° bends, respectively. Note that in the former case, Y-Y connection makes the entire waveguide channel. However, in the later, Y-Y connection is followed by a P-P connection. (**c**) and (**d**) Their traditional counterparts.

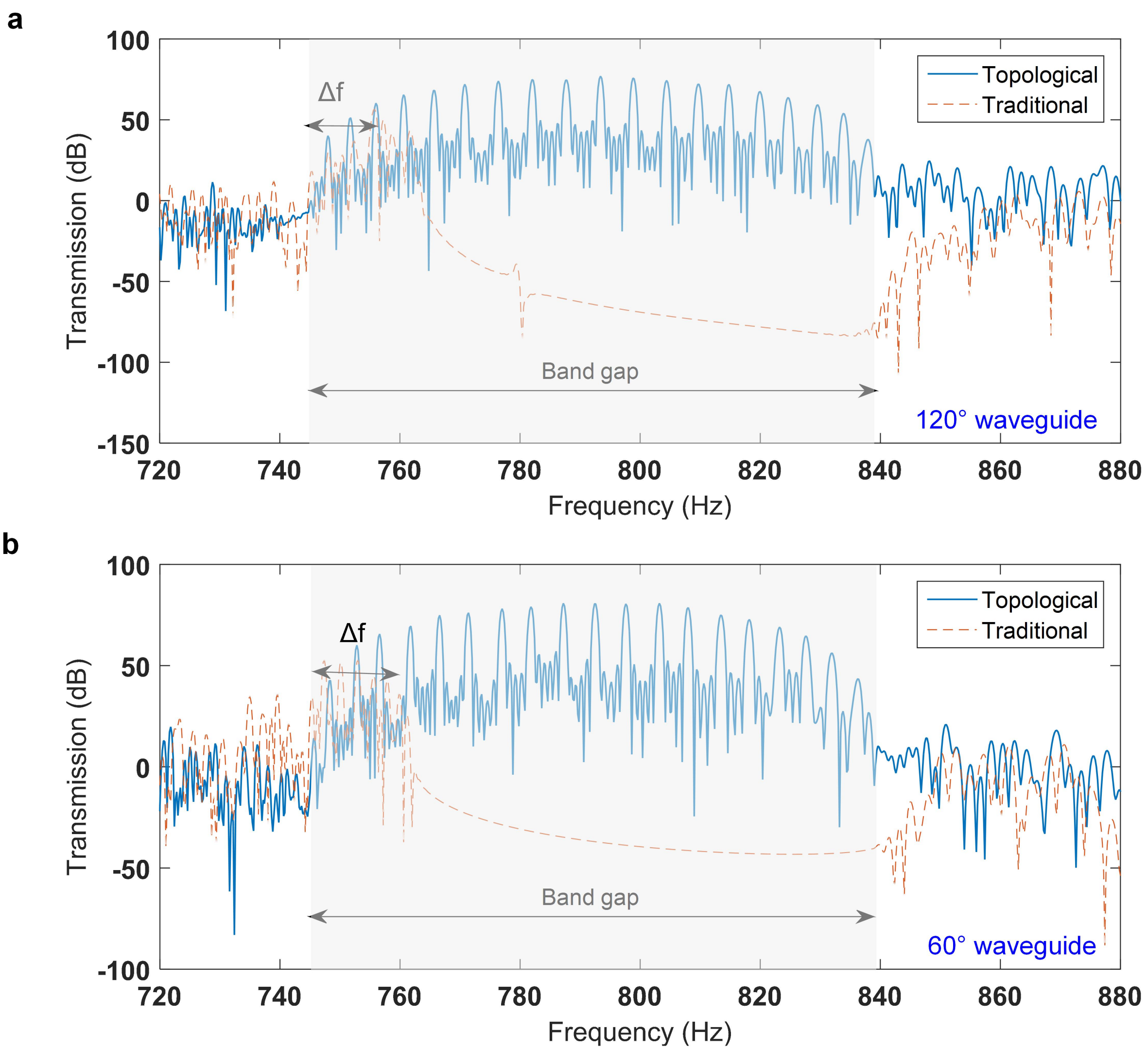

Supplementary Fig. 3: **Transmission of angle dependent waveguides.** (**a**) Comparing transmissions of topological and traditional waveguides for 120° bend. (**a**) The same for 60° bend.

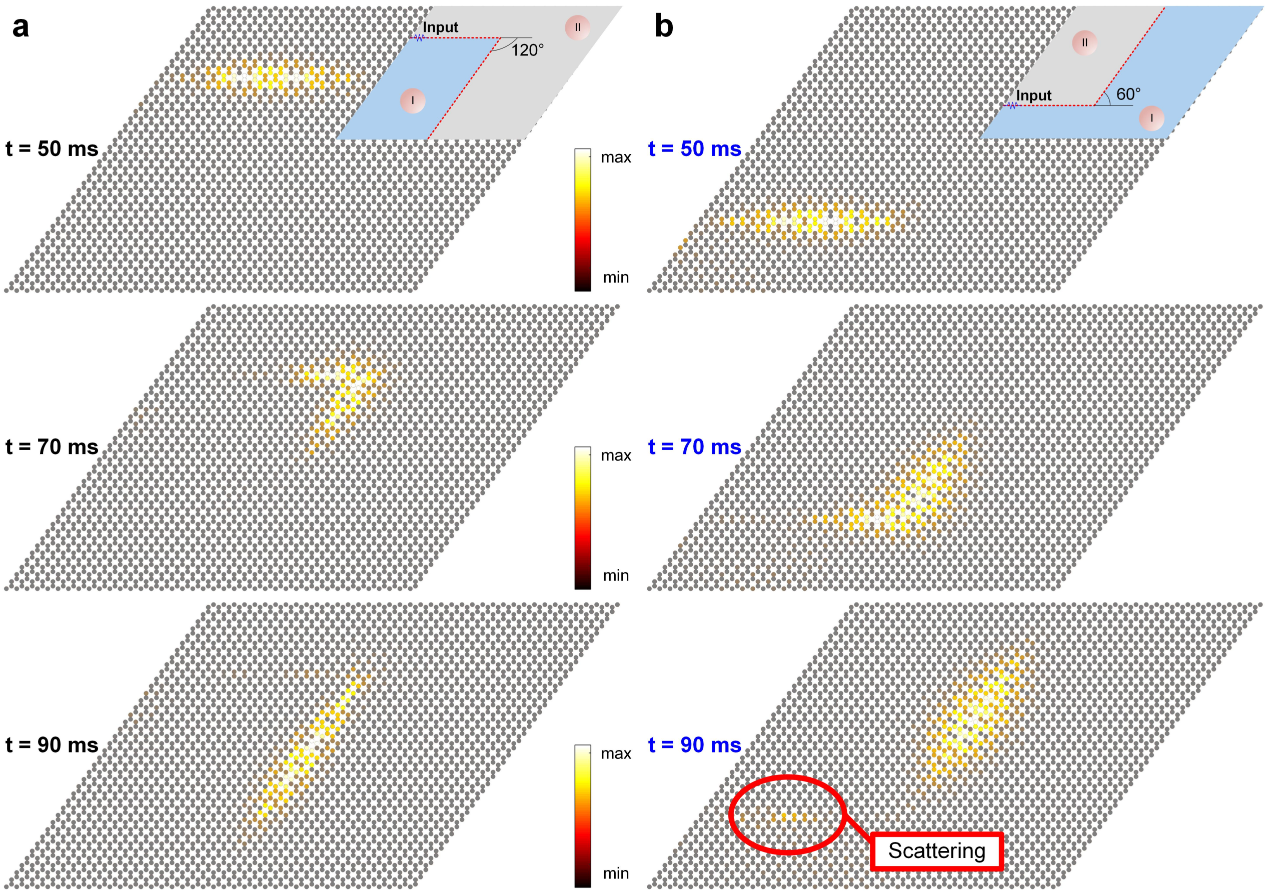

Supplementary Fig. 4: **Transient simulations for the topological waveguides**. (**a**) 120° waveguide. (**b**) 60° waveguide, for which, a slight scattering is highlighted at 90 ms. Colors denote the rotation of the bottom disks.
